# Systemic and cardiac susceptibility of immune compromised mice to doxorubicin

**DOI:** 10.1186/s40959-019-0037-6

**Published:** 2019-04-25

**Authors:** Amanda J. Favreau-Lessard, Hagen Blaszyk, Michael A. Jones, Douglas B. Sawyer, Ilka M. Pinz

**Affiliations:** 1 Maine Medical Center Research Institute, Center for Molecular Medicine, 81 Research Drive, Scarborough, ME 04074 USA; 2 Pathology Department, Maine Medical Center, 22 Bramhall St, Portland, ME 04102 USA; 3 Maine Medical Center, Cardiovascular Services, 22 Bramhall St, Portland, ME 04102 USA

**Keywords:** Anthracycline cardiotoxicity, Doxorubicin, Immune compromised mice

## Abstract

**Background:**

Anthracycline chemotherapy is an effective and widely used treatment for solid tumors and hematological malignancies regardless of its known cardiotoxicity. The mechanisms of the cardiotoxicity are not fully understood and methods to protect the heart during or following anthracycline chemotherapy are currently unclear. In order to examine the efficacy of human cell based therapy in anthracycline-induced injury, we characterized a mouse model using an immune compromised strain of mice capable of accepting human cells.

**Methods:**

Immune compromised mice (NOD.Cg-Prkdc^scid^ Il2rg^tm1Wjl^/SzJ) were repeatedly exposed to pharmaceutical grade doxorubicin (0.5 mg/kg – 4 mg/kg). Cardiotoxicity was assessed by echocardiography and μCT imaging of the coronary vascular bed as well as by flow cytometry and by histological assessments of anthracycline-induced cardiac tissue damage.

**Results:**

The immune compromised mice were highly susceptible to doxorubicin treatment. Doxorubicin induced both systemic and cardiac toxicities. Gastrointestinal and hepatic injury occurred at 4 mg/kg and 1.5 mg/kg dosing while mice receiving 0.5 mg/kg weekly only displayed hepatic damage. Repeated exposure to 0.5 mg/kg anthracyclines resulted in cardiac toxicity. Flow cytometric analysis of hearts indicated a loss in endothelial and cardiac progenitor cells after doxorubicin treatment. This endothelial loss is corroborated by the lack of small vessels detected by μCT in the hearts of mice exposed to doxorubicin. Histological assessment shows no overt cardiomyocyte injury but livers from mice treated with doxorubicin show marked hepatic plate atrophy with intracytoplasmic and canalicular cholestasis, rare pericentral hepatocellular necrosis and significant zone 3 iron accumulation, likely an indication of metabolic injury due to doxorubicin toxicity.

**Conclusions:**

Immune compromised mice are sensitive to doxorubicin therapy resulting in systemic complications in addition to cardiovascular toxicity. Anthracycline-induced cardiotoxicity is observed at very low doses in NOD.Cg-Prkdc^scid^ Il2rg^tm1Wjl^/SzJ mice.

## Background

Currently, there are over 15 million cancer survivors in the United States, which amounts to almost 5% of the country’s population [1]. This number is projected to double by 2040 [1]. Long-term survivors of cancer are faced with new challenges caused by cytotoxic cancer therapies. In particular, heart disease risk is increased in long-term cancer survivors treated with anthracyclines (i.e. Adriamycin also known as doxorubicin, Idarubicin, or Daunorubicin), a class of anti-tumor antibiotics that intercalate into DNA to inhibit synthesis thus inducing DNA damage and halting cell proliferation [2]. Anthracyclines are commonly administered as a therapeutic in adult and pediatric oncology due to its efficacy in treating a variety of solid and liquid based cancers such as leukemia, lymphoma, breast cancer, and lung cancer [3] regardless of the known side effects on the heart [4, 5].

In order to determine protective interventions for the heart due to anthracycline-induced cardiotoxicity (ACT), animal models of ACT have been developed that mirror the human disease. Many studies have investigated potential mechanisms of anthracycline-induced cardiac injury, to include myocyte death, increases in reactive oxygen species, and vascular damage [2]. Transgenic mouse models of ACT have been developed in a variety of mouse strains to investigate the role of a specific gene of interest [6–9]. It is difficult to identify a common mechanism of injury with the current published studies because the study design, dosing, length of treatment, severity of injury, and endpoints vary and therefore make comparisons between studies difficult.

More recently, cell therapies have been proposed to rescue the heart of individuals with anthracycline-induced cardiomyopathy. The lack of preclinical animal models examining these strategies may limit the success of clinical trials. The goal of our study was to develop a mouse model of ACT that mimics the human treatment regimen with repeated weekly anthracycline exposure, because the repeated stress and insult patients undergo may be a contributing factor for developing heart failure. In addition, we used an immune compromised mouse strain (NOD.Cg-Prkdc^scid^ Il2rg^tm1Wjl^/SzJ) to enable future cell-based therapeutic approaches using primary human derived cells in the setting of ACT.

## Methods

### Animals

Male and female NOD.Cg-Prkdc^scid^ Il2rg^tm1Wjl^/SzJ (NOD/Scid) mice were purchased from The Jackson Laboratory and housed in an AAALAC-accredited Clean Barrier Animal Facility. All animals and protocols were approved by Maine Medical Center Research Institute’s IACUC review committee. Pharmaceutical grade doxorubicin hydrochloride (2 mg/mL) was purchased in solution from Patterson Veterinary (Devens, MA). On the day of treatment, doxorubicin was diluted in 0.9% pharmaceutical grade saline for intraperitoneal injection. Mice were weighed and the doxorubicin dose was calculated based on body weight, thus injection volumes varied from 50 to 150 μL. Control mice received saline injections with volumes equivalent to the doxorubicin volume per body weight. The method of administration via intraperitoneal injection was selected after consulting with the Maine Medical Center Research Institute’s Animal Care and Use Committee as the safest and most consistent method of repeated injections of doxorubicin.

### Study design

A total of three studies were performed with different weekly doses (Table 1). Study I tested weekly injections of 4 mg/kg up to a cumulative dose of 8 mg/kg. Study II was designed as a dose escalation study to find the best tolerated dose. Study 3 then tested the best tolerated dose with repeated weekly injections to determine the timeframe for heart failure to be detected.

**Table 1:** Design of all three studies

Study	Weekly Doxorubicin Dose	Cumulative Doxorubicin Dose	# Saline Mice	# Doxorubicin Mice
I	4 mg/kg	8 mg/kg	14 (7♀, 7♂)	14 (7♀, 7♂)
II	Dose escalation: 0.5 mg/kg with weekly increases by 0.5 mg/kg	5 mg/kg	N/A	5 (3♂, 2♀)
III	0.5 mg/kg	3 mg/kg	4 (2♀, 2♂)	4 (2♀, 2♂)

### Flow cytometry

Hearts from mice were perfused with PBS containing heparin. Ventricular tissue was minced and incubated in a solution of Dispase II/Collagenase II/DNase I/CaCl_2_ at 37 °C for 20 min to collect a single cell suspension of non-myocyte cells from heart ventricles. To analyze differences in endothelial and progenitor cell populations, cell surface antibodies from BioLegend for CD45^APC/Cy7^, Sca1^PE/Cy7^, CD31^APC^ and CD105^PE^ were used. VioDye was utilized to stain for Live/Dead cells. All samples were run on the Miltenyi Biotec MACSQuant Cytometer. Analysis was performed using FlowJo V.10.0.8 software.

### Cardiac ultrasound

Echocardiography was performed with a Vevo2100 (VisualSonics), 40 MHz solid-state transducer, and the papillary muscle was used as orientation for the acquisition of short-axis M-mode images. Data were analyzed by the heart function package provided by VisualSonics Systems [10]. Diastolic and systolic LV internal dimensions were measured to evaluate LV structural changes. Fractional shortening and ejection fraction were calculated to determine changes in cardiac function [11].

### μCT imaging

Hearts were perfused with 25% Microfil (Flow Tech, Inc.) in the Langendorff mode. We used a low flow perfusion (500 μl/min) using a syringe pump (Harvard Apparatus) to prevent Microfil from entering the ventricular chambers. Hearts were suspended in PBS until the Microfil hardened. μCT imaging (Scanco VivaCT-40, Scanco Medical, Basserdorf, Switzerland) was performed at 10.5 μm resolution, with a voltage of 55kVp and a current of 145 μA, which created a 2048 × 2048 pixel image matrix. The tomograms were globally thresholded based on X-ray attenuation and used to render binarized 3-D images of the heart. The segmented image was analyzed with the standard Scanco morphology script to measure coronary diameter and volume. These data were presented as histograms.

### Histology

Heart and liver tissue were isolated from Study III animals and fixed in 10% formalin prior to histological processing (Maine Medical Center Research Institute Histology Core Facility). Tissues were embedded in paraffin and sectioned for immunohistological staining. Liver tissue was stained for Hematoxylin & Eosin (H&E), Masson trichrome, Reticulin, Iron, PAS and PAS-D. Cardiac tissue was stained for H&E and Masson trichrome. Routine histological methods were used. Images were acquired using a Zeiss Axioskop 40 microscope with a Canon EOS 60D camera at 4-, 10-, and 40-fold magnification.

### Statistics

All statistical analyses were performed using Prism (Version 7.03, GraphPad Software Inc). Specific analyses are listed in figure legends.

## Results

### Doxorubicin chemotherapy – systemic effects

**Fig. 1:**
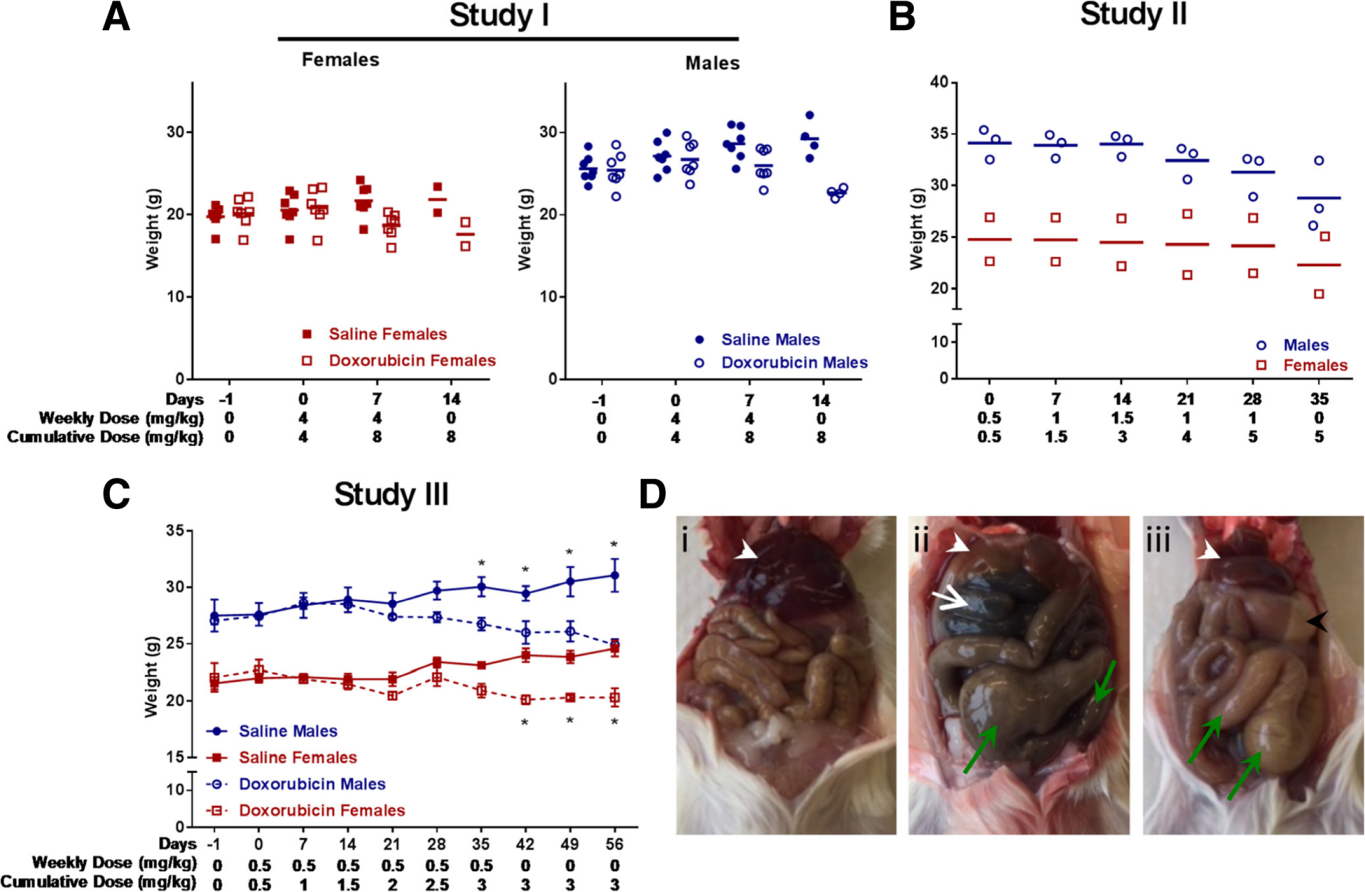
Body weights of saline and doxorubicin treated mice. **a** Study I with the highest dose of 4 mg/kg tested. Female mice treated with doxorubicin started to lose body weight after the first injection and continued to decline after the second injection. Male mice treated with doxorubicin had similar loss in body weight, however seemed to tolerate this dose slightly better than females. **b** In this dose escalation study, mice were treated starting with 0.5 mg/kg. The dose was increased weekly by 0.5 mg/kg. At a dose 1.5 mg/kg and a cumulative dose of 3 mg/kg, mice started to accelerate their loss in body weight and the weekly dose was reduced to 1.0 mg/kg. However, at a cumulative dose of 5 mg/kg the study had to be terminated because most mice had lost more than 10% of their body weight. **c** In study III mice received six weekly doses of 0.5 mg/kg for a cumulative dose of 3 mg/kg. At this dose mice tolerated doxorubicin treatment and the study continued for 8 weeks with moderate loss in body weights. **d** Representative in-situ photos of the abdominal cavity in (i) saline injected control mouse and (ii and iii) doxorubicin treated mice. Both doxorubicin treated mice have smaller livers compared to the saline treated mouse (white arrowheads), as well as air accumulation in the colon and intestines (green arrows). In (ii) parts of the intestines is necrotic (white arrow). The black arrow in (iii) shows the stomach that is visible due to the significantly smaller liver

To characterize anthracycline-induced cardiotoxicity in this specific mouse strain, we completed three separate studies with varying anthracycline dosages. Study I was designed with a dose of 4 mg/kg/week with a cumulative dose of not more than 24 mg/kg. This was based on literature findings, mostly from tumor studies, because cardiotoxicity studies are not routinely performed in this immune compromised strain. In the days following the first round of chemotherapy, all doxorubicin mice began exhibiting signs of distress and rapid weight loss (Fig. 1a). After the second round of chemotherapy all doxorubicin mice showed worsening signs of illness including rapid decline in body weight, diarrhea, ruffled fur, and hunching. The study was terminated early. Upon euthanizing mice, we observed severe gastrointestinal inflammation with pockets of air visible within the intestines, and some mice had areas of necrosis in their intestines, consistent with severe intestinal mucositis (Fig. 1d). In addition to the intestinal distress, we observed ascites and severe hepatotoxicity. These unanticipated effects appeared to account for the rapid decline in health, and not cardiac toxicity (Table 2).

**Table 2:** Study I echocardiography

				2 weeks			
		BASELINE		CONTROL		DOXORUBICIN	
Parameter	Unit	MEAN	SE	MEAN	SE	MEAN	SE
LVAW;d	mm	0.83	0.04	0.83	0.04	0.99*	0.15
LVAWS;s	mm	1.08	0.06	1.10	0.04	1.33*	0.15
LVID;d	mm	3.25	0.08	3.59	0.11	2.64*	0.22
LVID;s	mm	2.16	0.08	2.55	0.14	1.53*	0.24
LVPW;d	mm	0.79	0.05	0.92	0.08	1.04*	0.07
LVPW;s	mm	1.00	0.05	1.08	0.04	1.26*	0.11
Cardiac Output	ml/min	11.94	0.91	14.71	1.30	6.82*	1.32
Ejection Fraction	%	67.72	1.78	57.87	3.73	76.10	6.35
Fractional Shortening	%	36.94	1.33	30.05	2.51	45.31	6.28
Heart Rate	BPM	381	18	471	21	352	23
Stroke Volume	ul	30.62	1.56	31.30	2.33	18.91*	3.18
Volume;d	ul	45.62	2.74	54.64	4.53	26.40*	5.53
Volume;s	ul	15.00	1.47	23.34	3.47	7.49*	2.86
		*N* = 8 m, 4f		*N* = 4 m, 2f		*N* = 4 m, 2f	

*LV* left ventricle, *AW* anterior wall, *ID* internal diameter, *PW* posterior wall, *d* diastolic, *s* systolic, *BPM* beats per minute. *p < 0.05 repeated measures ANOVA

Based upon these findings, Study II was conducted to better characterize the dose tolerance and the maximum cumulative dose for this strain of mice. Mice received 0.5 mg/kg as a first dose and every week the dose increased by 0.5 mg/kg until the mice showed early signs of distress, such as modest loss of body weight (Fig. 1b). Doses of 0.5 mg/kg and 1.0 mg/kg were tolerated well. However, at the dose of 1.5 mg/kg (cumulative dose of 3 mg/kg) mice started to show a hunched posture. To reach our goal of repeated exposure to anthracyclines, we reduced the dose to 1 mg/kg for the final two rounds of chemotherapy. After reaching a cumulative dose 5 mg/kg, mice had lost 16.34 ± 9.634% of body weight, had ruffled fur and diarrhea. We determined this as the maximum cumulative dose this strain of mice can tolerate. This cumulative dose was also sufficient to induce cardiotoxicity (Table 3).

**Table 3:** Study II echocardiography

				5 weeks	
		BASELINE		DOXORUBICIN	
Parameter	Unit	MEAN	SE	MEAN	SE
LVAW;d	mm	0.88	0.08	0.70	0.09
LVAWS;s	mm	1.25	0.11	0.80*	0.04
LVID;d	mm	3.88	0.14	3.39	0.28
LVID;s	mm	2.62	0.21	2.61	0.27
LVPW;d	mm	0.89	0.03	0.79	0.08
LVPW;s	mm	1.17	0.06	0.89*	0.04
Cardiac Output	ml/min	20.44	2.46	10.77*	3.12
Ejection Fraction	%	62.50	5.03	49.80*	7.57
Fractional Shortening	%	33.60	3.43	25.20*	4.66
Heart Rate	BPM	488	17	400	49
Stroke Volume	μl	41.52	3.80	24.84*	5.53
Volume;d	μl	67.51	6.79	49.78	9.86
Volume;s	μl	26.00	5.35	24.94	6.24
		*N* = 3 m, 2f		*N* = 3 m, 2f	

*LV* left ventricle, *AW* anterior wall, *ID* internal diameter, *PW* posterior wall, *d* diastolic, *s* systolic, *BPM* beats per minute. *p < 0.05, paired t-test

We conducted Study III using a very low dose of 0.5 mg/kg repeated weekly injections. This low dose is roughly 1/20 the dose given to patients based off the Adriamycin product information [3] and 1/8 the dose used in many mouse studies [12]. Considering the results of Study II, we chose 3 mg/kg as the maximum cumulative dose. This regimen was tolerated well with male mice starting to slowly lose body weight after 4 weeks. This was in contrast to female mice, which were able to maintain their body weight until the end of the study after six injections (Fig. 1c). Both genders of mice were able to maintain their body weight after doxorubicin treatment stopped at 6 weeks and there was no apparent distress observed in any of the mice. Echocardiography was performed at the five-week time point and confirmed cardiotoxicity (Table 4). Two days prior to the endpoint at 8 weeks, all doxorubicin mice declined rapidly within 24 h of one another and either died or had to be euthanized due to severe distress.

**Table 4:** Study III echocardiography

				5 weeks	
		CONTROL		DOXORUBICIN	
Parameter	Unit	MEAN	SE	MEAN	SE
LVAW;d	mm	0.90	0.11	0.79	0.06
LVAWS;s	mm	1.18	0.05	1.04	0.07
LVID;d	mm	2.98	0.17	3.50*	0.16
LVID;s	mm	1.69	0.14	2.50*	0.22
LVPW;d	mm	1.03	0.14	0.81*	0.09
LVPW;s	mm	1.40	0.12	1.10*	0.07
Cardiac Output	ml/min	15.2	1.0	16.3	0.8
Ejection Fraction	%	79.8	3.8	60.0*	6.2
Fractional Shortening	%	47.5	3.7	31.8*	4.1
Heart Rate	BPM	556.6	29.4	484.3	11.7
Stroke Volume	ul	27.4	2.0	33.6*	1.3
Volume;d	ul	34.6	3.8	57.0*	4.7
Volume;s	ul	7.3	2.1	23.4*	5.7
		*N* = 2 m, 2f		*N* = 2 m, 2f	

*LV* left ventricle, *AW* anterior wall, *ID* internal diameter, *PW* posterior wall, *d* diastolic, *s* systolic, *BPM* beats per minute. *p < 0.05 paired t-test

### Cardiac ultrasound

Cardiac morphology and function were assessed in all three studies (Tables 2-4). In study I, mice showed changes in ventricular morphology that are consistent with cardiac edema. Wall thickness increased in anterior and posterior walls of the left ventricle, causing diastolic and systolic left ventricular volumes to decrease.

In study II, we observed changes in cardiac function and morphology that are consistent with heart injury induced by ACT (Table 3). After 5 weeks of treatment at 3 mg/kg cumulative dose (echocardiography was performed prior to the 5th injection) all doxorubicin treated mice show thinning of left ventricular walls, lower ejection fraction as well as decreased cardiac output. These data suggest that for this strain of NOD/Scid mice, a cumulative dose of 3 mg/kg is sufficient to induce ACT.

**Fig. 2:**
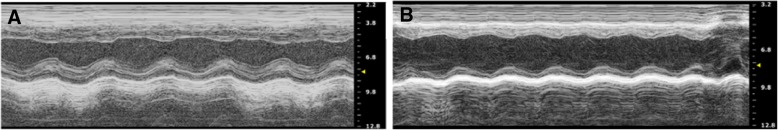
Representative M-mode echocardiograms after 5 weeks of doxorubicin treatment in study III. **a** M-mode echocardiogram at baseline and **b** after 5 weeks of weekly 0.5 mg/kg doxorubicin injections

In study III, this treatment regimen increased systolic volumes in the heart in NOD/Scid mice already after 5 weeks and a cumulative dose of 2 mg/kg (the week 5 echocardiography was performed prior to the 5th injection) as well as a decrease in fractional shortening compared to the control mice (Table 4). Figure 2 compares representative M-mode images from baseline vs. doxorubicin treated hearts showing the increased systolic volume.

### Non-myocyte flow cytometry analysis of hearts

**Fig. 3:**
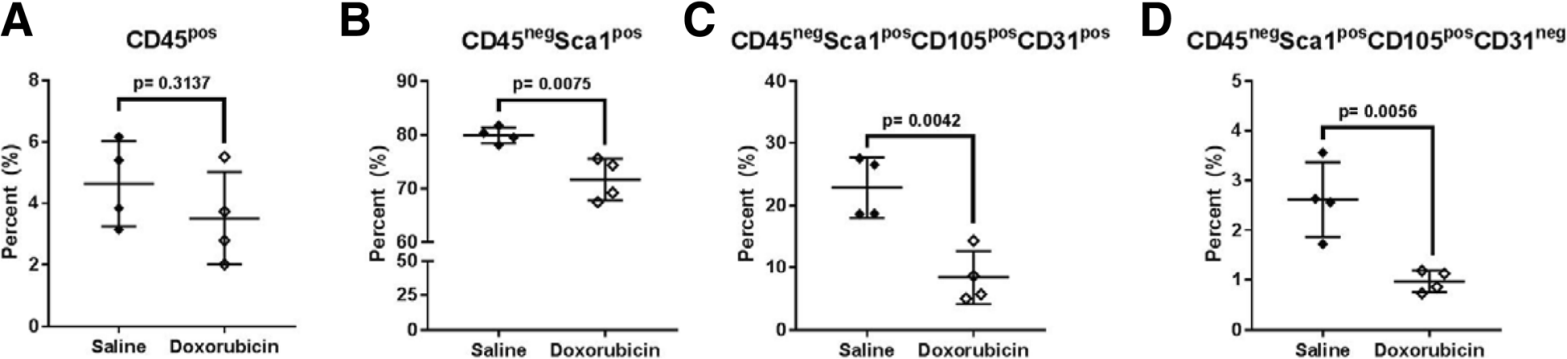
Cardiac progenitor cell analysis following doxorubicin exposure in hearts from study I. **a** CD45^pos^ immune cells were not different between groups. **b** CD45^neg^Sca1^pos^ non-immune cardiac cells were reduced in hearts with doxorubicin treatment. **c** CD45^neg^Sca1^pos^CD105^pos^CD31^pos^ endothelial cell numbers are more than 50% lower in doxorubicin treated hearts. **d** CD45^neg^Sca1^pos^CD105^pos^CD31^neg^ cardiac progenitor cells are significantly decreased in hearts exposed to doxorubicin. Individual data points with mean and SD are shown. *N* = 4, t-test with *p* values indicated

Flow cytometric analysis of non-myocyte cell populations in ventricular tissue was performed of hearts from mice in Study I (*n* = 4/treatment). Non-myocyte cells were stained with a mixture of antibodies to identify immune cells and markers of endothelial and cardiac progenitor cells. Dead cells were excluded and live cells were analyzed for the presence of the pan-immune marker, CD45. We observed no significant difference in CD45 expression between saline and doxorubicin treated mice (Fig. 3a). Within the CD45 negative non-immune cell population, cells were analyzed for Sca1 expression, a cell surface marker on both cardiac progenitor and endothelial cells. Doxorubicin treated mice had a significant decrease in CD45^neg^Sca1^pos^ cells compared to the saline treated mice (Fig. 3b). Further analysis of the CD45^neg^Sca1^pos^ population was performed for CD31 and CD105 expression to distinguish the endothelial and cardiac progenitor populations. Doxorubicin significantly decreased both endothelial (CD45^neg^Sca1^pos^CD105^pos^CD31^pos^) and cardiac progenitor (CD45^neg^Sca1^pos^CD105^pos^CD31^neg^) populations compared to the saline treated mice (Fig. 3c-d). The loss of endothelial cells suggests damage to the vasculature of the heart. Furthermore, the loss of resident cardiac progenitor cells may suggest that these mice do not have the local progenitor cell pool to repair anthracycline-induced damage in the heart following completion of the chemotherapy regimen.

### μCT assessment of heart vasculature

**Fig. 4:**
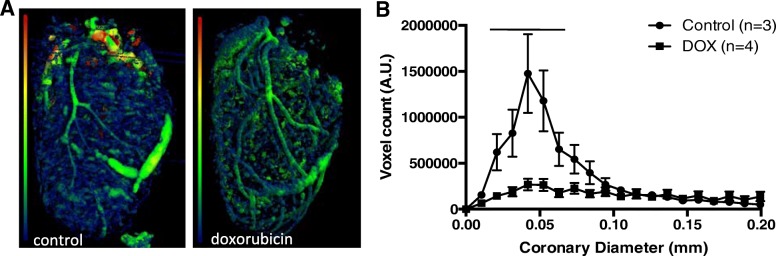
Quantification of coronary blood vessels by μCT in hearts from study I and III. **a** Representative μCT images of Microfil perfused hearts. Saline injected control hearts show more of the smaller (dark blue) coronaries compared to doxorubicin exposed hearts. **b** Quantification of coronary diameters in saline and doxorubicin treated hearts. Saline treated hearts have a larger number of coronaries with smaller diameters compared to hearts from doxorubicin treated mice. MEAN ± SE, *n* = 3–4, multiple t-test comparison with Sidak-Bonferroni correction for multiple comparisons, line indicates *p* < 0.05

Previous studies have shown that anthracycline chemotherapy causes vascular injury [13]. The mechanism of injury is thought to be via reactive oxygen species. To determine whether vascular injury is present in our mouse model of ACT, a subset of hearts from the first and third study underwent perfusion with Microfil and subsequent coronary vascular density measurements by μCT. Mouse hearts exposed to doxorubicin lose coronaries with smaller diameters (Fig. 4). These data suggest endothelial damage.

### Liver and heart histological analysis

**Fig. 5:**
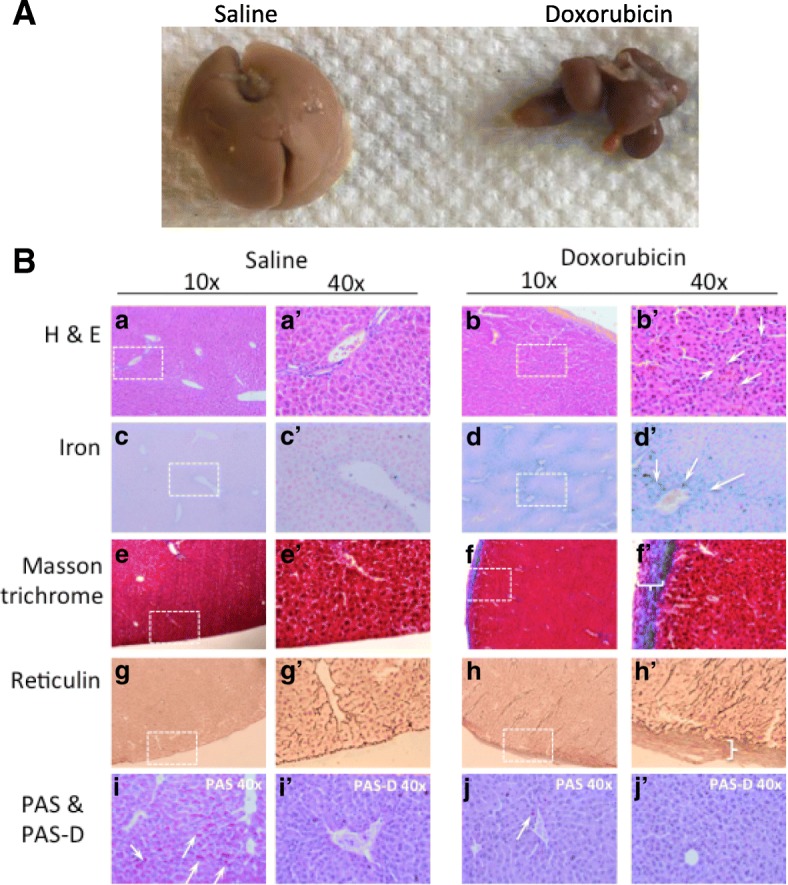
Liver morphology and histology of saline and doxorubicin injected NOD/Scid mice. **a** Gross morphology of the liver from saline and doxorubicin injected mice. The liver from the doxorubicin-injected mouse was the worst case observed, however varying degrees of liver atrophy were observed in all animals from all three studies. **b** a, a’, b and b’: H & E stain of liver showing hemosiderin deposits (b’, brown staining/white arrows) in the doxorubicin exposed liver. c, c’, d and d’: Iron staining confirms deposition of hemosiderin (d and d’, blue stain/white arrows) throughout the liver in doxorubicin treated mice. e, e’, f and f’: Masson trichrome staining reveals a thickened fibrous capsule in doxorubicin treated mice (f’, white brackets). g, g’, h and h’: Reticulin staining confirms a thick fibrous cap in doxorubicin treated mice (h’, white bracket). i, i’, j and j’: PAS and PAS-D staining to show the presence or absence of glycogen stores. Saline treated mice show red staining in their livers (i, white arrows), which disappears with dispase treatment (i’, PAS-D). Doxorubicin treated mice do not show staining with PAS, suggesting depleted hepatic glycogen stores (j and j’). Representative images shown from study III mice

One of the most striking observations was the clear differences in the liver size and structure at tissue harvest. The doxorubicin treated mice had significantly smaller livers and hepatic structures appeared abnormal due to their smaller size. Histologically, there was marked hepatic plate atrophy with diminished cytoplasmic volume and both intracellular and canalicular cholestasis (Fig. 5). Fatty change or significant fibrosis was not seen. Native bile ducts appeared intact and there were no features of venous outflow obstruction. Only occasional hepatocellular dropout was evident, largely in pericentral distribution. Interestingly, there was significant iron accumulation, again predominantly pericentral (Fig. 5c and d). All of the above features point to metabolic/toxic liver injury that has been ongoing and is likely a direct effect of doxorubicin toxicity.

**Fig. 6:**
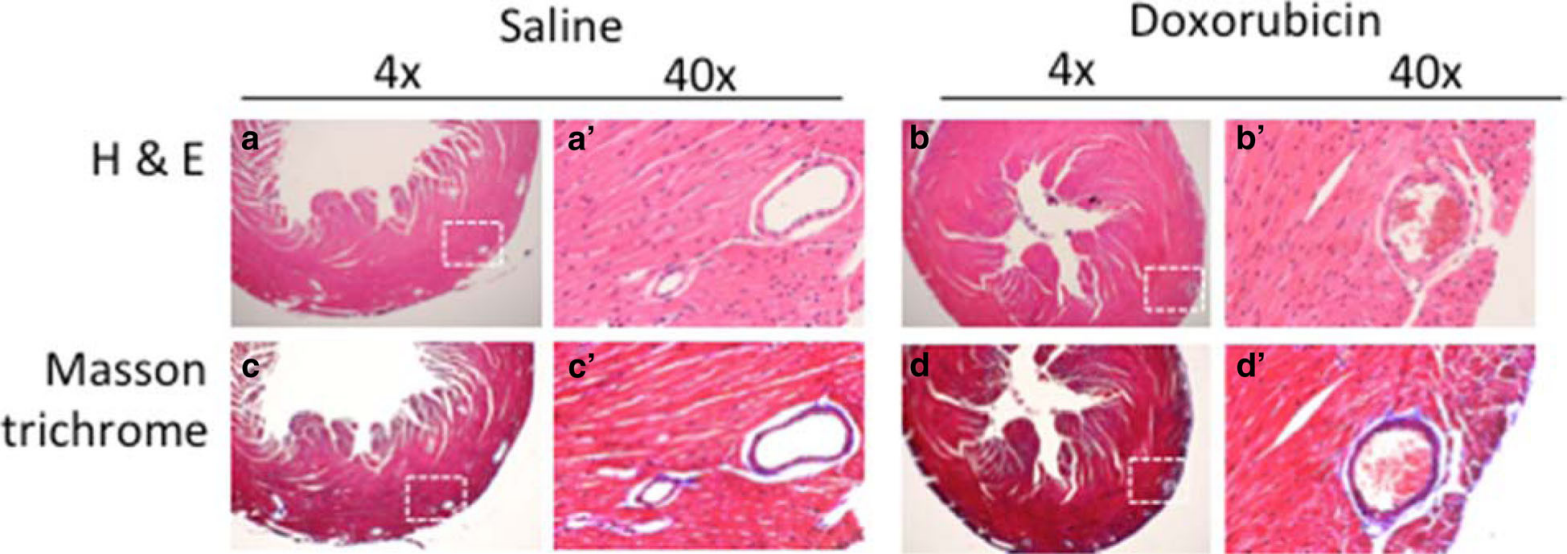
Cardiac histology of saline and doxorubicin injected NOD/Scid mice. a, a’, b and b’: H & E staining showing normal morphology in both groups of mice. Cardiomyocyte structure appears to be unchanged with doxorubicin treatment in these conditions. c, c’, d and d’: Masson trichrome staining does not show increased fibrosis in doxorubicin treated hearts. Also, coronary blood vessels (c’ and d’) do not appear different between groups. Representative images from study III hearts

A subset of hearts was collected for histological assessment, specifically staining for Hematoxylin and Eosin (for structure and morphology) and Masson trichrome (for evidence of damage and fibrosis). Cardiac structure and morphology comparing the saline and doxorubicin treated mice were normal with no obvious disruption in cardiomyocytes (Fig. 6). Additionally, there was no increase in Masson trichrome staining in saline or doxorubicin hearts indicating no increased fibrosis or damage (Fig. 6).

## Discussion

The purpose of this study was to develop a model of anthracycline-induced cardiotoxicity in an immune compromised mouse strain. The use of the NOD/Scid strain has not been characterized in this injury model and is essential for investigating the efficacy of human cell based therapies as a potential treatment for this type of cardiac injury. Over the course of this study, we determined that these mice have both systemic and cardiovascular susceptibility to anthracyclines. In addition to the cardiac toxicity, there was evidence of hepatic and gastrointestinal toxicity contributing to the decline of global health in the mice. Our results suggest that the mechanism of anthracycline-induced cardiac injury is potentially based on several different mechanisms and has a strong dependence on dosages used.

### Differences to other anthracycline studies in mice

We have found a large variation in published studies using anthracyclines in mice regarding strain, dosing, total cumulative dose and dose timing, dependent on the goals of the experiment. Doses ranged from 2.5 mg/kg of body weight [14] to as high as 8 mg/kg [15]. Some doses were administered once, typically at a high dose, while other studies treated with multiple doses but the time between doses varied widely. In addition, when immune compromised mouse strains were used, these studies focused on cancer research and not on chemotherapy-related cardiotoxicity. Another interesting observation is that most studies utilize non-pharmaceutical grade doxorubicin that is purchased lyophilized, which requires reconstitution and is then used at various doses. For this study, we chose pharmaceutical grade doxorubicin that did not require reconstitution, thus eliminating batch-to-batch variations in concentrations and we used the same grade that is being used in patients. However, we found that we needed much lower concentrations compared to studies performed by others.

### Systemic effects vs. cardiac effects – dosing considerations

In our first two studies in NOD/Scid mice, we have found profound systemic toxicity of doxorubicin. The most highly affected organ systems were the intestines and the liver. This indicates that in NOD/Scid mice the gastrointestinal toxicity of doxorubicin is very pronounced and that these effects very likely contributed to the rapid decline of the mice in these studies. In study III, we used a very low dose of doxorubicin in order to avoid the toxic systemic effects. Mice tolerated this very low dose without any signs of distress and had only modest body weight loss. As mentioned, 2 days prior to completion of the time course all mice declined rapidly and either died or had to be euthanized. We did not observe intestinal mucositis in these mice, but all mice showed signs of hepatic toxicity. The observed histologic changes can be entirely attributed to metabolic liver toxicity of doxorubicin. This is true both globally, as the observed, significant liver cell atrophy appeared rather diffuse in distribution, as well as zonal along the cytochrome P450 enzyme activation distribution within the liver. The observed iron accumulation is likely a secondary phenomenon due to a metabolic compromise, mirroring the more acute hepatocellular damage found predominantly in zone 3.

In addition to the hepatic toxicity, this last cohort showed signs of cardiac toxicity at the 5 week time point. For these mice, it is difficult to determine if the liver toxicity or the cardiac toxicity was responsible for their decline. In other studies investigating ACT, the systemic effects that we have observed have not been mentioned, raising the question whether this is a strain specific effect.

The disrupted immune system in this NOD/Scid strain causes a lack of adaptive immunity and impairs the innate immunity due to natural killer (NK) cell deficiency and impaired T and B cells. Given the high susceptibility to systemic toxicity, the lack of the adaptive immune response may play a role in this injury model. The Scid mutation results in the B and T cell deficiency due to mutation in the Prkdc protein, which is involved in the DNA repair processes [16]. In addition, the IL2rg mutation hinders multiple interleukin signaling cascades and thus results in a lack of functional NK cells [16]. There is limited knowledge on the adaptive immune response in the context of anthracycline use but the role of the adaptive immune response has been investigated in the context of ischemic cardiac injury. The adaptive immune response is shown to play pivotal roles in aiding the innate immune response following ischemic cardiac injury [17]. Studies performed in other models of cardiac injury (such as a myocardial infarction model) may lend insight into the role of the adaptive and innate immune responses in an anthracycline-induced cardiotoxicty model as well as the systemic susceptibility the mice in our study demonstrated. Further investigation in the immune response may elucidate mechanisms contributing to the systemic and cardiac toxicity caused by anthracycline therapy.

### Discussion of potential mechanism – endothelial vs. cardiomyocyte

Despite the systemic side effects and the significant hepatic toxicity, the results of our study suggest that the cardiac insult from anthracyclines may not solely be caused by cardiomyocyte toxicity as suggested by other studies [2]. While we have not found damage to the cardiomyocytes based on histological assessment, we can not exclude some of the previously suggested mechanisms such as disruption to sarcomere structure [18] or oxidative stress to cardiomyocytes [19]. Our study suggests that as a further injury mechanism, the cardiac endothelium seems to be a primary target of the toxic chemotherapeutic effects. The more than 50% loss of cardiac endothelial cells and the significant lower number of small diameter coronary blood vessels support this conclusion. The loss of endothelial cells can alter vessel permeability and increased permeability can result in increased inflammatory infiltration and alter contractility of the myocardium [20, 21]. In study I via flow cytometry, we found no significant differences in CD45^pos^ immune cells after 2 weeks of doxorubicin treatment but this may change if we looked at a later time point in the study after chemotherapy treatment is completed since the immune system can be altered during chemotherapy administration [22]. In addition, the loss of cardiac progenitor cells may predispose these hearts to a long-term loss of cardiac repair potential. Cardiac progenitor cells are considered a reserve pool to aide in repair of the damaged cells within the heart. Cardiac progenitor cells are capable of differentiating into mature cardiac myocytes, endothelial cells, and fibroblasts. Loss of this pool would limit the heart’s capability to replace the cells lost following chemotherapy exposure. This may provide one explanation why some cancer survivors develop heart failure years after completion of their chemotherapy.

Preclinical models to investigate the efficacy of cell-based therapy for treating anthracycline-induced cardiac injury in cancer survivors are needed. With this study identifying a model of vascular toxicity instead of cardiac myocyte toxicity, we have a model to further test the efficiency of cell-based therapies to restore endothelial cell number and vascular function. Our group has previously isolated highly proliferative cells from epicardial left ventricular biopsies (eHiPCs) in patients undergoing coronary artery bypass grafting surgery [23]. We identified a subset of these eHiPCs that exhibit endothelial-like properties and are currently undergoing further characterization to determine their potential for revascularization. These cells may prove to be quality candidates to test in future studies as a therapy for the vascular toxicity associated with ACT.

## Conclusions

In summary, we have established an immune compromised mouse model for doxorubicin-induced cardiovascular toxicity using NOD/Scid mice by administering very low doses of pharmaceutical grade doxorubicin. Utilizing this model in NOD.Cg-Prkdc^scid^ Il2rg^tm1Wjl^/SzJ mice may allow us to investigate the efficacy of progenitor cell based therapies to alleviate established cardiotoxic damage specifically to vessels and progenitors. It should be recognized that close observation is required with this strain, specifically checking on these mice for signs of gastrointestinal distress, lethargy, dehydration, and loss of appetite. At study endpoint, examining mice for intestinal complications and liver toxicity should be performed and noted as these could contribute to the phenotype. Any such complications on a systemic level should be considered when drawing conclusions even though the main purpose of the study is to focus on the cardiac damage from anthracyclines. This study provides a basis to explore cell-based treatment options for long-term cancer survivors with chemotherapy-induced heart failure to overcome, the often fatal, consequences of cytotoxic cancer therapies.

## Abbreviations

ACT: Anthracycline-induced cardiotoxicity
AW: Anterior wall
BPM: Beats per minute
d: diastolic
H&E: Hematoxylin and Eosin
ID: Internal diameter
LV: Left ventricle
NK: Natural killer
NOD/Scid: NOD.Cg-Prkdc^scid^ Il2rg^tm1Wjl^/SzJ
PW: Posterior wall
s: systolic
